# Feasibility of Adsorption Kinetic Models to Study Carrier-Mediated Transport of Heavy Metal Ions in Emulsion Liquid Membranes

**DOI:** 10.3390/membranes12010066

**Published:** 2022-01-03

**Authors:** Gerardo León, Elisa Gómez, Beatriz Miguel, Asunción María Hidalgo, María Gómez, María Dolores Murcia, María Amelia Guzmán

**Affiliations:** 1 Departamento de Ingeniería Química y Ambiental, Universidad Politécnica de Cartagena, Paseo Alfonso XIII 52, 30206 Cartagena, Spain; beatriz.miguel@upct.es (B.M.); maguzmanmv@gmail.com (M.A.G.); 2 Departamento de Ingeniería Química, Campus de Espinardo, Universidad de Murcia, 30100 Murcia, Spain; egomez@um.es (E.G.); ahidalgo@um.es (A.M.H.); maria.gomez@um.es (M.G.); md.murcia@um.es (M.D.M.)

**Keywords:** emulsion liquid membranes, carrier-mediated transport, adsorption, kinetic, mechanism

## Abstract

Emulsion liquid membranes have been successfully used for the removal of different types of organic and inorganic pollutants by means of carrier-mediated transport mechanisms. However, the models that describe the kinetics and transport of such mechanisms are very complex due to the high number of model parameters. Starting from an analysis of the similarity between the elemental mechanisms of carrier-mediated transport in liquid membranes and of transport in adsorption processes, this paper presents an experimental analysis of the possibility of applying kinetic and mechanistic models developed for adsorption to carrier-mediated transport in emulsion liquid membranes. We study the removal of a target species, in this case, Cu(II), by emulsion liquid membranes containing membrane phase solutions of benzoylacetone (carrier agent), Span 80 (emulsifying agent) and kerosene (diluent), and hydrochloric acid as a stripping agent in the product phase. The experimental results fit the pseudo-second-order adsorption kinetic model, showing good relationships between the experimental and model parameters. Although both Cu(II) diffusion through the feed/membrane interface boundary layer and complex Cu-benzoylacetone diffusion through the membrane phase controls Cu(II) transport, it is the former step that mainly controls the transport process.

## 1. Introduction

The presence of heavy metals, elements with atomic weights of between 63.5 and 200.6, and a specific gravity greater than 5.0 [1], in industrial effluents and wastewater, is a serious environmental problem since they are non-biodegradable in natural conditions, have a tendency to accumulate in living organisms, causing a variety of diseases and disorders, and have an inhibiting effect on the biodegradation of organic pollutants which may also be present in wastewater [2,3]. Therefore, their concentrations should be reduced to acceptable levels before being discharged into the environment. Many industries are responsible for the direct or indirect discharge of heavy metals into the environment: mining, hydrometallurgy, textiles, tanneries, pulp and paper, pesticides, petrochemicals, refining, batteries, fertilizers, electroplating, among others [4,5].

Over the past few years, several techniques have been described for removing heavy metal ions wastewater, among them chemical precipitation [6], cementation [7], coagulation-flocculation [8], adsorption [9,10,11,12], electrochemical processes [13,14], and photocatalytic methods [15]. 

Since the 1970s, when Bhattacharyya et al. [16] and Sato et al. [17] used ultrafiltration and reverse osmosis membranes, respectively, for treating metal plating wastewaters, several membrane-based processes have been used to remove heavy metal ions from wastewaters, including micellar enhanced ultrafiltration [18,19], nanofiltration [20,21], reverse osmosis [22], forward osmosis [23], electrodialysis [24] and polymer inclusion membranes [25].

Liquid membranes have also been used for the efficient removal of heavy metal ions from aqueous solutions [26,27,28,29,30]. A liquid membrane system consists of two miscible liquids (feed and product phases) separated by a third liquid (immiscible in both) as a membrane phase. The efficiency of metal ion removal by liquid membrane processes is significantly improved through the use of so-called carrier-mediated transport [31]. In such transport, a reagent (carrier agent) is incorporated into the membrane phase to transport the heavy metal ion from the feed to the product phase through the membrane phase. At the feed/membrane interface, the carrier and the heavy metal ion reversibly form a compound that is soluble in the membrane phase. This compound is transported through the membrane phase due to its concentration gradient and is broken at the membrane/product interface by reacting with a stripping agent; the heavy metal ion is released into the product phase and, as a consequence of its concentration gradient, the carrier diffuses to the opposite interface through the membrane phase before initiating a new separation cycle.

There are three basic types of liquid membranes: bulk, supported, and emulsion liquid membranes [32]. In emulsion liquid membranes, a primary emulsion formed by the membrane phase and the product phase is dispersed in the feed phase, the encapsulated internal droplets of the product phase and the external feed phase being separated by the membrane phase.

**Figure 1:**
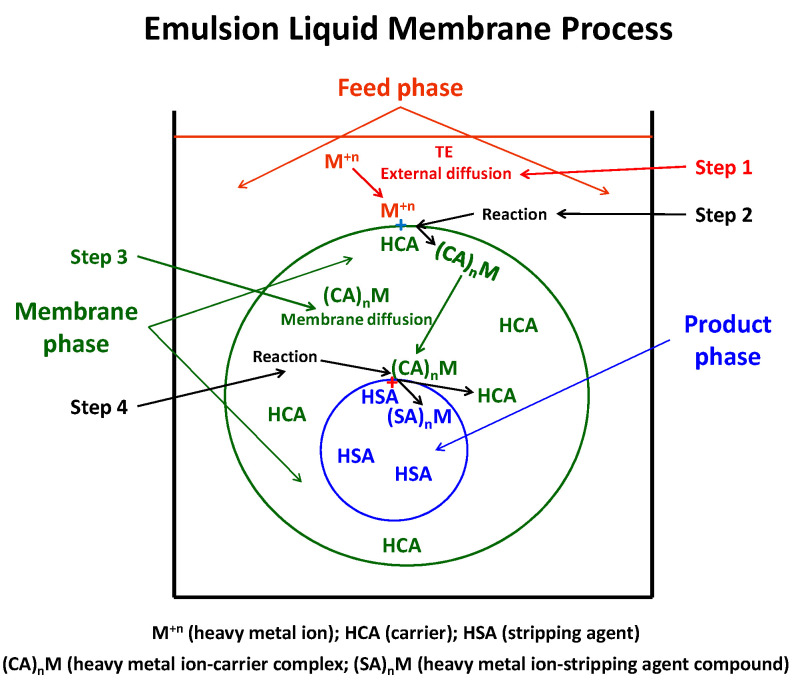
Mechanism of heavy metal ion permeation through an emulsion liquid membrane by carrier-mediated transport.

The elementary mechanism of heavy metal ion permeation through an emulsion liquid membrane by a carrier-mediated transport includes four basic steps (Figure 1) [33]:

Step 1. Heavy metal ion diffusion through the boundary layer of the feed phase to the feed/membrane interface.
Step 2. Heavy metal ion reaction, at the feed/membrane interface, with the carrier present in the membrane phase forming a metal-carrier complex soluble in the membrane phase.
Step 3. Complex diffusion, in the membrane phase, from the feed/membrane interface toward the membrane/product interface.
Step 4. Complex breakdown at the membrane/product interface due to the reaction between the heavy metal ion and stripping agent present in the product phase and carrier regeneration.

The models described in the literature to study the kinetics and mechanism of carrier-mediated transport in emulsion liquid membranes tend to be highly complex as they include a large number of model parameters [33,34,35]. It must be taken into account that from using models to fit experimental data, it is necessary to make simplifying assumptions and that all theoretical models are approximated, but some of them are useful [36]. For this reason, it may be of interest the look for simpler mathematical models to which the experimental results are adequately adjusted, allowing the explanation for the phenomenon under study.

**Figure 2:**
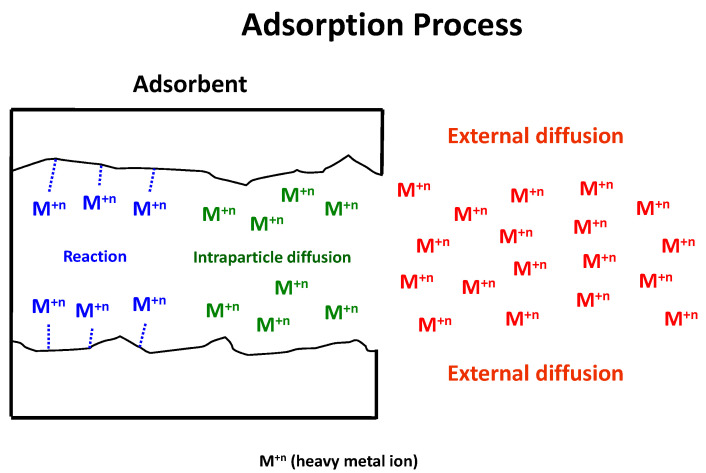
Mechanism of the adsorption of an adsorbate onto an adsorbent.

Bearing this in mind, let us highlight the similarity that the above described carrier-mediated transport mechanism in emulsion liquid membranes has with the elemental mechanism of adsorption of an adsorbate onto an adsorbent, which includes three basic steps (Figure 2) [37,38]:

Step 1. External diffusion (film diffusion), which is the transport of the heavy metal ion from the bulk phase to the external surface of the adsorbent.
Step 2. Intraparticle diffusion (pore diffusion), which is the transport of the heavy metal ion from the external surface into the pores.
Step 3. Surface reaction, which is the attachment of the heavy metal ion to the internal surface of the sorbent.

As the reaction steps are much faster than those of diffusion, the transport kinetics in a carrier-mediated emulsion liquid membrane process will be governed by diffusion of the metal ion through the boundary layer of the feed phase to the feed/membrane interface or by diffusion of the metal-complex in the membrane phase. In the same way, in an adsorption process, the transport kinetics will be governed by external diffusion or by intraparticle diffusion.

This great similarity between the transport mechanisms of both processes makes the kinetic and mechanistic study of the carrier-mediated transport in emulsion liquid membrane processes by means of the kinetics and mechanistic models developed for adsorption processes an interesting option for experimental analyses, especially because no such study seems to have been carried out to date.

Accordingly, in this paper, we analyze the carrier-mediated transport of Cu^2+^ (as target species) through emulsion liquid membranes using benzoyl acetone (carrier), kerosene (diluent), and Span 80 (emulsifying agent) in the membrane phase and hydrochloric acid as stripping agent in the product phase, in different experimental conditions, and fitting the obtained results to kinetic and mechanistic models that have been described for adsorption processes.

## 2. Materials and Methods

### 2.1. Materials

Benzoylacetone (99%), kerosene, and Span 80 (sorbitan monooleate) were supplied by Sigma Aldrich; copper (II) chloride (98.5%), hydrochloric acid (37%), acetic acid (96%), and sodium acetate 3-hydrate (for analysis) were obtained from Panreac.

### 2.2. Methods

The internal aqueous phase consisted of an aqueous solution of hydrochloric acid ranging from 0.05 M to 0.50 M. The organic membrane phase was prepared by dissolving the appropriate amounts of the carrier (benzoylacetone, 0.1% to 2.0%) and the surfactant (Span 80, 5%) in kerosene (organic diluent) by gently mixing using a magnetic stirrer. 

The water in oil primary emulsion (*w*/*o*) was prepared by mixing the aqueous product phase with the organic membrane phase, at a volume ratio of 1/1, under stirring, before emulsifying the mixture at a high stirring rate (2700 rpm) for 5 min to obtain a stable emulsion, using an OMNI MIXER homogenizer (Omni International, Kennesaw, GA, USA). This emulsion was dispersed into the external feed Cu(II) aqueous phase (0.025 M in acetate buffer, pH ranging from 4.0 to 5.5) in a cylindrical glass cell to form the secondary emulsion (*w*/*o*/*w*), at a 1/2 emulsion phase/feed phase volume ratio. The content of the glass cell was stirred (50 to 200 rpm) in order to disperse the *w*/*o* emulsion in the external phase to form the *w*/*o*/*w* double emulsions. 

The external feed phase was periodically sampled and, after settling for 3 min to separate feed and emulsion phases, the Cu(II) content in the feed phase was analyzed by atomic absorption spectrometry, using a ContrAA 700 (Analytik Jena, Edinburgh Instruments, Livingston, UK) instrument at a wavelength of 324.8 nm. 

The duration of the experiments was 20 min to ensure that no increase occurred in the concentration of Cu(II) in the feed phase with time as a consequence of emulsion breakage in any of the studied experimental conditions.

Experiments were conducted in duplicate at room temperature, the results showing a maximum deviation of 3%. No removal of Cu(II) from the feed phase was detected in the absence of a carrier agent in the membrane phase.

Analysis of the efficiency of an elimination process using emulsion liquid membranes is usually carried out using three parameters: removal percentage, flux, and permeability.

The percentage of Cu(II) removal from the feed phase (RP) was determined according to Equation (1), where C_f,0_ and C_f,t_ are the initial and time t concentrations of Cu(II) in the feed phase.

(1) $$\mathrm{RP}=\frac{C_{f,0}-C_{f,t}}{C_{f,t}}\times 100$$

Initial apparent fluxes (J_a_) and apparent permeabilities (P_a_) of Cu(II) through the feed/membrane interface were obtained from the plots against the time of C_f,t_ and ln(C_f,t_/C_f,0_), respectively, during the first five minutes of the experiment, according to Equations (2) and (3), in which it was assumed that membrane area is proportional to the emulsion volume (A = k·V_emul_), that the stripping reaction is fast enough so that there is no accumulation of Cu(II) in the membrane phase and that the same preparation conditions of the emulsion lead to the same size uniformity of the emulsion globules [27,39].

(2) $$J_{a}=-\frac{V_{f}\cdot {\mathrm{dC}}_{f}}{V_{\mathrm{emul}}\cdot \mathrm{dt}}$$

(3) $$\ln\frac{C_{f,t}}{C_{f,0}}=-\frac{V_{\mathrm{emul}}\cdot P_{a}\cdot t}{V_{f}}$$

where V_f_ and V_emul_ are the volumes of feed and of emulsion, respectively.

Additionally, the initial Cu(II) removal rate (V_0,exp_) was obtained by extrapolating to zero (intercept) the representation of the Cu(II) removal rate (mg_Cu(II)remov_/L_emul_·t) against time during the first three minutes of the experiment.

In an adsorption process, the parameter adsorption capacity is defined for any time t (q_t_) or at equilibrium (q_e_), as the quantity of adsorbate (usually expressed in mg) retained per mass (or volume) unit of adsorbent (m/g or mg/L, respectively) [37,38]. 

In the case of emulsion liquid membranes, we will define this parameter as the amount of compound removed from the feed phase (expressed in milligrams) per volume unit of emulsion phase (expressed in liters).

Accordingly, the amount of Cu(II) removed from the feed phase per volume unit of emulsion phase, at any time t, q_t_ (mg/L), and at equilibrium, q_e_ (mg/L), was estimated from the relationships Equations (4) and (5), where C_f,e_ is the equilibrium concentration (at 20 min) of Cu(II) in the feed phase (mg/L).

(4) $$q_{t}=\left(C_{f,0}-C_{f,t}\right)\cdot \frac{V_{f}}{V_{\mathrm{emul}}}$$

(5) $$q_{e}=\left(C_{f,0}-C_{f,e}\right)\cdot \frac{V_{f}}{V_{\mathrm{emul}}}$$

The kinetics of the carrier-mediated transport of Cu(II) through emulsion liquid membranes was analyzed using four adsorption kinetic models: Lagergren pseudo-first-order, Ho pseudo-second-order, Elovich and Avrami.

The linear form of the Lagergren pseudo-first-order model [40] is expressed by Equation (6). The values of the model constants, q_e,pfo_ (mg/L), and k_pfo_ (pseudo-first-order rate constant, min^−1^) can be obtained from the plot of ln(qe − qt) vs. time.

(6) $$\ln\left(q_{e,\exp}-q_{t}\right){=\mathrm{lnq}}_{e,\mathrm{pfo}}-k_{\mathrm{pfo}}\cdot t$$

Equation (7) shows the linearized form of the Ho pseudo-second-order kinetic model [41]. The values of the model constants, q_e,pso_ (mg·L^−1^), k_pso_ (pseudo-second-order rate constant, L·mg^−1^·min^−1^), and V_0,pso_ (initial adsorption rate, defined as k_pso_·(q_e,pso_)^2^, mg·L^−1^·min^−1^) can be obtained by plotting t/qt against time.

(7) $$\frac{t}{q_{t}}=\frac{1}{k_{\mathrm{pso}}\cdot q_{e,\mathrm{pso}}^{2}}+\frac{t}{q_{e,\mathrm{pso}}}$$

The Elovich kinetic model [42] is generally expressed, assuming that α·β·t > 1, by Equation (8). The values of the model constants, α (initial adsorption rate, mg·L^−1^·min^−1^) and β (number of available sites for adsorption, L·mg^−1^), can be obtained from the plot of q_t_ versus lnt.

(8) $$q_{t}=\frac{\ln(\alpha \cdot \beta )}{\beta }+\frac{\ln t}{\beta }$$

The linear form of the Avrami kinetic model [43] is usually described by Equation (9). The values of model constants K_av_ (Avrami’s constant rate, min^−1^) and n_av_ (Avrami’s order model) can be obtained from the plot of ln[−ln(1 − q_t_)] versus lnt.

(9) $$\ln\left[-\ln\left({1-q}_{t}\right)\right]{=\mathrm{lnK}}_{\mathrm{av}}{+n}_{\mathrm{av}}\cdot \mathrm{lnt}$$

The above described kinetic models do not provide information about the rate-controlling step of the carrier-mediated transport of Cu(II) through an emulsion liquid membrane, which is characterized, as mentioned above, by Cu(II) diffusion through the boundary layer of the feed/membrane interface (equivalent to the external diffusion in an adsorption process), by Cu-carrier complex diffusion through the membrane phase (equivalent to the intraparticle diffusion in an adsorption process), or by both. To obtain information about that rate-controlling step, Weber and Morris intraparticle diffusion and Boyd adsorption models were used.

The Weber and Morris intraparticle diffusion model [44] is commonly expressed by Equation (10). The values of the model constants k_intp_ (intraparticle diffusion rate constant, mg·L^−1^·min^−1/2^) and C_i_ (effect of extraparticle diffusion, mg·L^−1^) can be obtained by plotting q_t_ against t^1/2^.

(10) $$q_{t}{=k}_{\mathrm{intp}}\cdot t^{1/2}{+C}_{i}$$

When that plot is linear and passes through the origin, intraparticle diffusion is the rate-controlling step of the adsorption process. Otherwise, both extraparticle and intraparticle diffusion steps are involved in controlling the rate. 

If the latter occurs, it is necessary to identify which of these two steps is the one that mainly controls the rate of the overall process, usually using for this purpose the Boyd kinetic model [45], which is expressed in the form:

(11) $$\mathrm{Bt}=-0.4977-\ln\left(1-\frac{q_{t}}{q_{e}}\right)$$

If the plot of Bt versus time is a straight line and passes through the origin, the adsorption process is mainly controlled by the intraparticle diffusion step; otherwise, it is mainly controlled by the extraparticle diffusion step.

## 3. Results and Discussion

### 3.1. Kinetics of Cu(II) Transport

**Figure 3:**
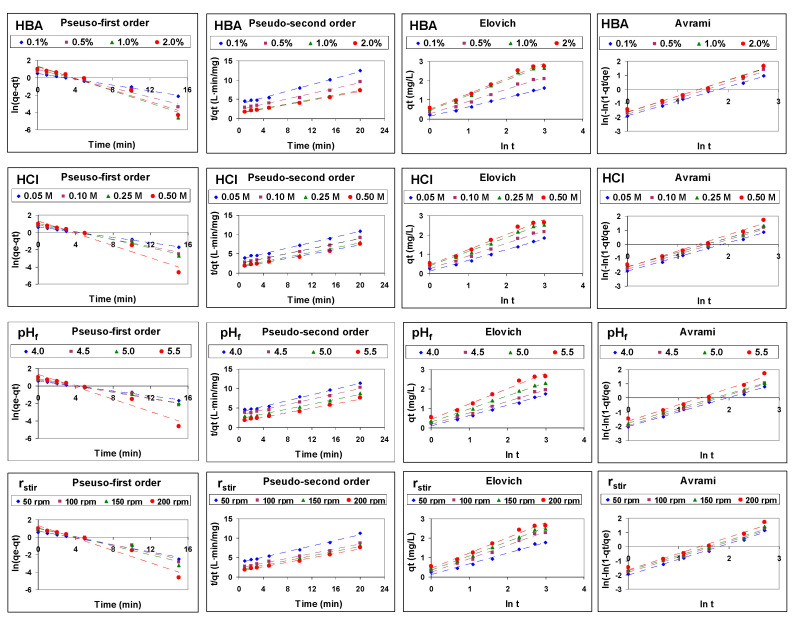
Fitting of the data obtained in the different experimental conditions to the studied kinetic models.

The fitting of the data to these models in the different studied experimental conditions is shown in Figure 3. The reliability of the fit was determined based on the values of the determination coefficient (R^2^).

Data of the carrier-mediated transport of Cu(II) through emulsion liquid membranes, in the different experimental conditions studied, are best fitted by the pseudo-second-order kinetic model, which showed determination coefficient values higher than 0.99 (0.9903–0.9975) in all cases, while pseudo-first-order (0.9478–0.9984), Elovich (0.9816–0.9957) and Avrami (0.9812–0.9985) models provided several determination coefficients lower than 0.99.

The pseudo-second-order was originally proposed for the removal of divalent metal cations from water using zeolites, assuming adsorption/reaction mechanisms [46]. The model assumes that the uptake rate is second order with respect to the available sites [37] and describes adsorption processes in which the chemical bonding between adsorbates and functional groups on the surface of adsorbents is responsible for the adsorption capacity of the adsorbent [45,47]. This is what occurs in the process of Cu(II) removal by carrier-mediated transport in emulsion liquid membranes, which includes a stripping reaction, that takes place at the membrane/product interface to release the metal ion into the permeate phase. In this reaction, each Cu(II) ion is striped in the product phase by a reaction with two HCl molecules.

In addition, it is known that the pseudo-second-order kinetic adsorption model closely fits the experimental results when the initial concentration of the species to be removed is low, the experiment time range included in the fit is broad, and there are many active sites to react with the species to be removed [38]. These three aspects occurred in our study of Cu(II) removal by emulsion liquid membranes using carrier-mediated transport.

Therefore, the pseudo-second-order model is the one that best describes all the possible steps included in the global transport mechanism, with a k value that is a result of a complex interplay between different controlling mechanisms, although no information is provided concerning the mass transfer mechanism. 

From an analysis of the results obtained from fitting the experimental data to the pseudo-second-order kinetic model, several conclusions can be drawn that support the feasibility of the use of adsorption kinetic models to study the carrier-mediated transport of heavy metal ions through emulsion liquid membranes.

**Figure 4:**
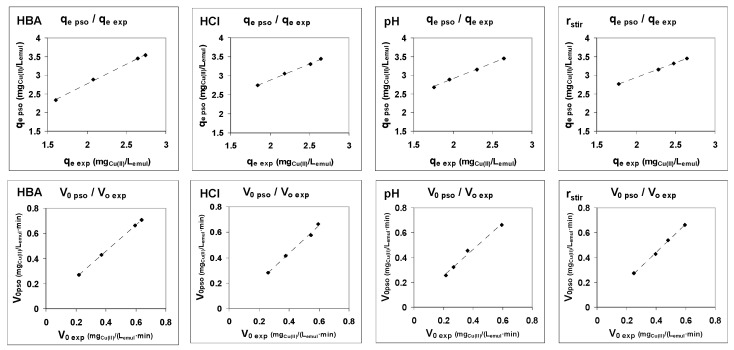
Relationship between experimental and pseudo-second-order model values of parameters q_e_ and V_0_.

First, as can be seen from Figure 4, there is a good relationship between the values of the theoretical parameters of the pseudo-second-order model (q_e,pso_ and V_0,pso_) and the corresponding experimental values of these parameters (with coefficients of determination that range between 0.983 and 0.997)

**Figure 5:**
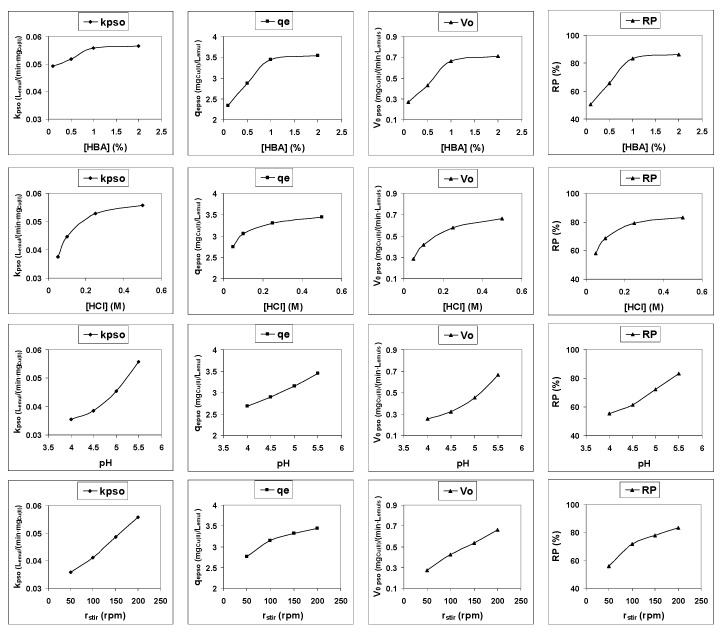
Variation in the values of pseudo-second-order model parameter (k_pso_, q_e,pso_, V_0,pso_) and those of the removal percentage (RP) with the different experimental conditions studied.

Second, the variation of the values of these theoretical parameters of the pseudo-second-order model with the values of the different experimental parameters studied follows a trend that is practically similar to that which follows the experimentally calculated removal percentage of Cu(II) from the feed phase (Figure 5). As the removal percentage is usually used as a parameter to analyze the influence of the different experimental conditions on the efficiency of an emulsion liquid membrane process, any of the three parameters obtained from fitting of the experimental results to the pseudo-second-order kinetic model can also be used to analyze this influence.

Accordingly, using both the elimination percentage and any of the three parameters of the model (k_pso_, q_e,pso_ or V_0,pso_), it can be observed that the efficiency of the process increases as the HBA concentration in the membrane phase increases from 0.1% to 1%, above which it remains constant; the efficiency increases as the HCl concentration in the product phase increases from 0.05 M to 0.50 M, the efficiency increases as the pH of the feed phase increases from 4.0 to 5.5 and the efficiency increases as the stirring rate of the secondary emulsion increases from 50 rpm to 200 rpm.

The increase in benzoylacetone concentration in the membrane phase from 0.1% to 1.0% favors the removal of Cu(II) from the feed phase due to the greater number of Cu(II) ions that can form the complex Cu-benzoylacetone, which increases its concentration gradient through the membrane phase, favoring its diffusion to the membrane/product interface. At higher benzoylacetone concentrations, this effect is partially neutralized by the increase in the emulsion viscosity, which leads to an increase in the emulsion globules size and consequently to a decrease in the mass transfer surface area, leading to removal efficiencies that remain constant.

The increase in both the concentration of HCl in the product phase and the pH in the feed phase increase the gradient of the proton concentration between the product and the feed phases, which is the driving force of the Cu(II) transport process, leading to an increase in the Cu(II) removal efficiency.

The increase in the stirring rate leads to the increase in the number of smaller emulsion globules, leading to an increase in the mass transfer surface area and consequently to an increase in Cu(II) removal efficiency.

**Figure 6:**
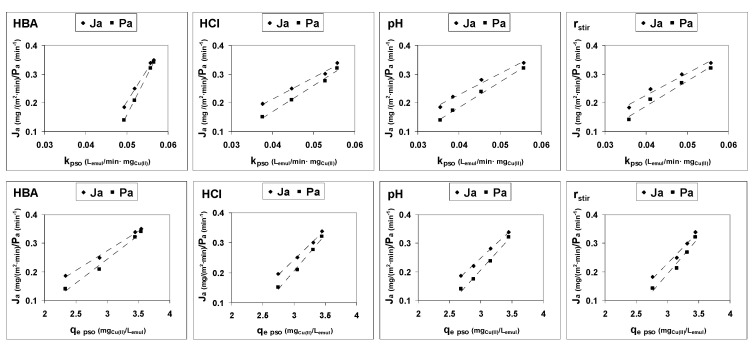
Relationship between the values of pseudo-second-order model parameters (k_pso_ and q_e,pso_) and the experimental initial apparent fluxes (Ja) and permeabilities (Pa).

Thirdly, there is a good linear relationship between the parameters of the model and the values of the initial apparent fluxes and permeabilities calculated from the experimental results, as can be seen in Figure 6.

### 3.2. Mechanism of Cu(II) Transport

**Figure 7:**
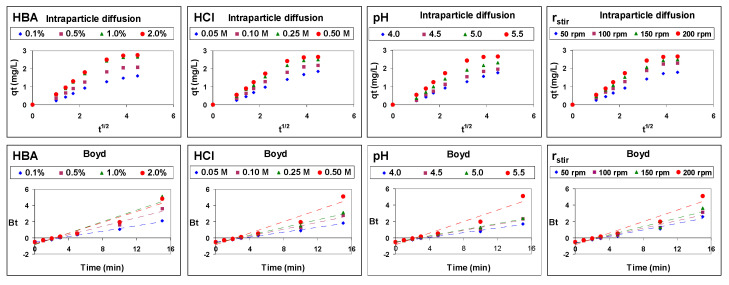
Analysis of the experimental data of carrier-mediated transport of Cu(II) through emulsion liquid membranes by intraparticle diffusion and Boyd models.

The carrier-mediated transport of Cu(II) through emulsion liquid membranes was analyzed by the Weber-Morris and Boyd mechanistic models to obtain information about the rate-controlling step of the carrier-mediated transport of Cu(II) through an emulsion liquid membrane (Figure 7).

Intraparticle diffusion model representations are not linear over the entire time range. The existence of a clear multilinearity means that Cu-benzoylacetone complex diffusion in the membrane phase (from the feed/membrane interface toward the membrane/product interface) is not the only rate-controlling step, but rather that more than one step is involved. That is, the dual nature of the intraparticle model graphs confirms that both Cu(II) diffusion through the boundary layer of the feed/membrane interface and Cu-benzoylacetone complex diffusion through the membrane phase control the Cu(II) transport from the feed phase to the product phase. 

As Boyd model representations are not linear and they do not pass through the origin, it can be deduced that Cu(II) diffusion through the boundary layer of the feed phase to the feed/membrane interface is the step that mainly controls the rate of the carrier-mediated transport process of Cu(II) through an emulsion liquid membrane. 

## 4. Conclusions

We have studied the possibility of applying kinetic and mechanistic models developed for the adsorption process to the carrier-mediated transport in emulsion liquid membranes by analyzing Cu(II) removal from aqueous solutions (pH 4.0–5.5) by emulsion liquid membranes containing solutions of benzoylacetone (carrier agent, 0.5–2.0%) and Span 80 (emulsifying agent, 5%) in kerosene, as membrane phase, and hydrochloric acid (0.05 M–0.50 M) as stripping agent in the product phase. The membrane phase/product phase volume ratio used was 1/1, the feed phase/emulsion phase volume ratio was 2/1, and the stirring rate ranged from 50 to 200 rpm. The experimental results were analyzed by four kinetic adsorption models, the best fit being obtained with the pseudo-second-order kinetic model (R^2^ > 0.99). A good relationship between the experimental parameters and those of the model was observed, and it was verified that variations in these model parameters with the different experimental conditions follow the same trend as that of the removal percentage calculated experimentally, the parameter normally used to analyze the efficiency of the liquid membrane process. Likewise, a linear relationship was observed between the model parameters and the initial apparent fluxes and permeabilities determined experimentally. The study of the transport mechanism suggests that although both Cu(II) diffusion through the feed/membrane interface boundary layer and complex Cu-benzoylacetone diffusion through the membrane phase control Cu(II) transport, it is the former step that mainly controls the transport process.

## Data Availability

Please exclude this statement. The study did not report any data.
